# Quantification of Proliferating and Mitotically Active Retinal Cells in Mice by Flow Cytometry

**DOI:** 10.21769/BioProtoc.5024

**Published:** 2024-07-05

**Authors:** Hope K. Vanzo-Sparks, Sarah E. Webster, Mark K. Webster, Cindy L. Linn

**Affiliations:** Western Michigan University, Department of Biological Sciences, Kalamazoo, MI, USA

**Keywords:** Neurogenesis, Flow cytometry, Retina, PNU-282987, Alpha7 nAChR agonist, Müller glia, Retinal ganglion cells, Photoreceptors, Proliferation, Mitosis

## Abstract

Adult mammals lack the ability to regenerate retinal neurons after injury. However, in previous studies from this lab, topical application of the selective alpha7 nicotinic acetylcholine receptor (nAChR) agonist, PNU-282987, has been associated with an increase in the number of retinal neurons in adult murine models both in the presence and absence of injury to the retina. Additionally, studies assaying mitotic markers have shown a substantial increase in the amount of mitotically active and proliferating cells with the topical application of the alpha7 nAChR agonist. However, these previous studies were performed using fluorescent immunolabeling and subsequent confocal microscopy, thus limiting the number of antibodies that can be multiplexed. As a result, we have developed a flow cytometry method that allows for the multiplexing and analysis of multiple external and internal markers in dissociated retinal cells. In this paper, a step-by-step protocol is described for the labeling of multiple retinal cell types such as retinal ganglion cells, rod photoreceptors, and Müller glia, concurrently with Müller glia–derived progenitor cells that arise after treatment with PNU-282987.

Key features

• Neurogenesis in the adult mammalian retina.

• Flow cytometry of retinal cells.

• PNU-282987-induced mitotic activity in the retina.

• Dissociation of the retina for flow cytometry analysis.

Graphical overview

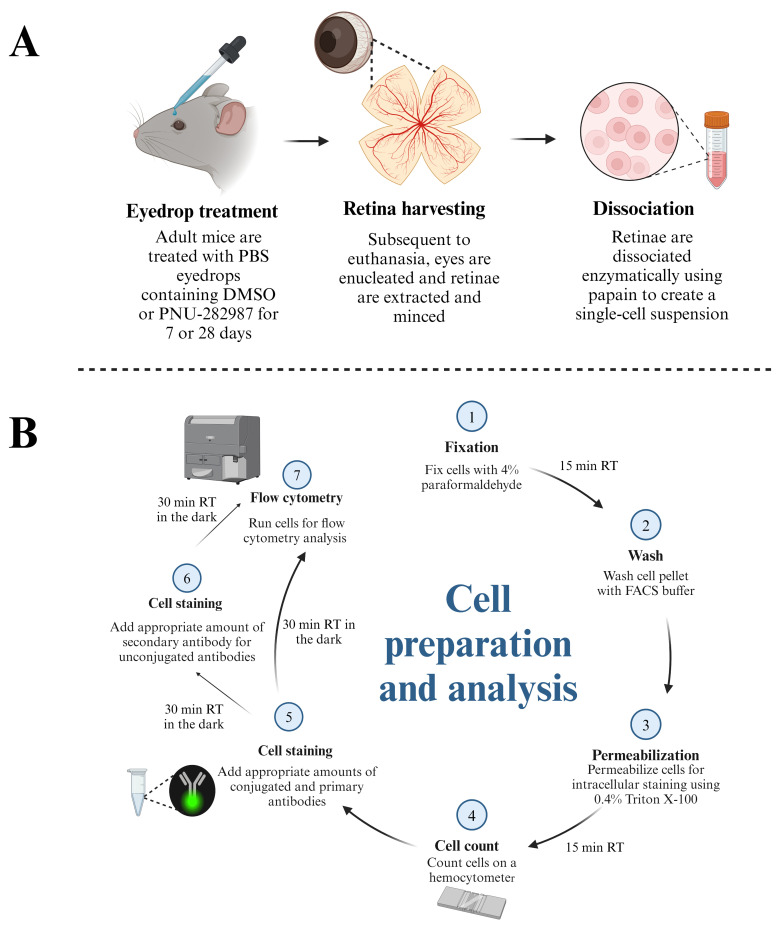

**Schematic demonstrating the protocol for preparation of retinal cells for flow cytometry analysis.** (A) Adult mice (3–6 months) are subjected to topical PBS eyedrop treatment containing DMSO (control groups) or PNU-282987 (experimental groups). Both eyedrop treatments contain 1 mg/mL of BrdU to label proliferating cells. After treatment, mice are euthanized, and retinae are harvested for dissociation using papain. (B) Dissociated retina cells are fixed and permeabilized before aliquots are taken for cell counts on a hemocytometer. After determining the number of cells present, conjugated antibodies and unconjugated primary antibodies are added at the appropriate dilutions. Fluorescent secondary antibodies are added for markers that are unconjugated. Cells are then subjected to flow cytometric analysis using a BD LSRFortessa.

## Background

Adult mammals cannot typically regenerate retinal neurons after injury [1–3]. However, previous research from this lab using adult mice has shown that the selective alpha7 nicotinic acetylcholine receptor (nAChR) agonist, PNU-282987, can induce neurogenesis in adult mammals when applied as eyedrops in the presence or absence of any retinal injury [4–10]. PNU-282987 is believed to act on alpha7 nAChRs in the retinal pigment epithelium to release signaling molecules onto the end feet of Müller glia to introduce cell cycle re-entry. From there, Müller glia de-differentiate and form retinal progenitor cells that eventually develop into mature retinal neurons [6,9]. However, these studies were performed using fluorescent immunolabeling and subsequent confocal microscopy, thus restricting the number of antibodies that can be multiplexed and limiting the examination of cells undergoing mitosis and proliferation. As a result, we have developed a flow cytometry method that allows for the multiplexing and analysis of multiple external and internal markers in dissociated retinal cells. In this paper, a step-by-step protocol is described for the labeling of multiple retinal cell types such as retinal ganglion cells, rod photoreceptors, and Müller glia, concurrently with mitotically active and proliferating cells that arise after treatment with PNU-282987.

## Materials and reagents

1. Papain dissociation system, which includes papain, DNase I, ovomucoid inhibitor, and EBSS (Worthington Biochemical Corporation, catalog number: LK003150)
2. Phosphate buffered saline (PBS) 10× concentrate (Sigma-Aldrich, catalog number: P5493)
3. PNU-282987 hydrate (N-[(3R)-1-azabicyclo[2.2.2]oct-3-yl]-4-chlorobenzamide hydrochloride) (Sigma-Aldrich, catalog number: P6499)
4. BrdU (5-Bromo-2’-deoxyuridine) (Sigma-Aldrich, catalog number: 19-160)
5. Fetal bovine serum (FBS) (Thermo Fisher Scientific, catalog number: A5670701)
6. Paraformaldehyde (PFA) (Sigma-Aldrich, catalog number: P6148)
7. Hydrochloric acid (HCl) (Sigma-Aldrich, catalog number: 258148)
8. Sodium hydroxide (NaOH) (Sigma-Aldrich, catalog number: S5881)
9. Triton X-100 (Sigma-Aldrich, catalog number: T8787)
10. Dimethyl sulfoxide (DMSO) (Sigma-Aldrich, catalog number: D8418)
11. BD Horizon^TM^ brilliant stain buffer (BD Biosciences, catalog number: 563794)
12. Fc receptor binding inhibitor polyclonal antibody, eBioscience^TM^ (Thermo Fisher Scientific, catalog number: 14-9161-73)
13. Fluorescent antibodies and unconjugated blocking antibodies (see Table 1)
14. UltraComp eBeads^TM^ compensation beads (Invitrogen, catalog number: 01-2222-42)
  **Table 1.** Antibody panel used in this protocol

Antibody	Fluorophore	Clone	Host	Company	Catalog number	Dilution (per 1 × 10^6^ cells)
Rhodopsin	Unconjugated	RET-P1	Mouse	Invitrogen	MAS-11741	3 μL
Zenon kit	Alexa-405	-	-	Invitrogen	Z25013	-
Thy 1.2	APC-eFluor-780	53-2.1	Rat	eBioscience	47-0902-82	1 μL
Vimentin	Biotinylated	280618	Rat	R&D Systems	BAM2105	2 μL
Streptavidin	BUV-395	-	-	BD Biosciences	BD 564176	2 μL
Ki67	BUV-737	20Raj1	Mouse	eBioscience	367-5699-42	5 μL
BrdU	PerCP-eFluor-710	BU20A	Mouse	eBioscience	46-5071-42	1 μL

**Solutions**

1. DMSO/PNU-282987/BrdU eyedrop solution (see Recipes)
2. DMSO/BrdU eyedrop solution (see Recipes)
3. 4% PFA (see Recipes)
4. FACS buffer with 4% FBS (see Recipes)
5. 0.4% Triton X-100 (see Recipes)
6. 0.1 M HCl (see Recipes)

**Recipes**

1. **DMSO/PNU-282987/BrdU eyedrop solution**
**Caution:** Appropriate PPE must be worn when handling chemicals.
  Reconstitute 50 mg of PNU-282987 in 1.66 mL of DMSO to make a 100 mM stock that can be stored at 4 for several months. Dissolve 5 mg of BrdU in 5 mL of DMSO to make a stock solution of 100 mg/mL BrdU that can be stored at -20 for several months. To make PNU-282987/BrdU eye drop solution, mix 9.8 mL of 1× PBS, 100 μL of 100 mM PNU-282987, and 100 μL of BrdU to achieve a final solution of 1 mg/mL BrdU and 1 mM PNU-282987. Store at 4 for one month.
2. **DMSO/BrdU eyedrop solution**
  Mix 9.8 mL of 1× PBS, 100 μL of DMSO, and 100 μL of BrdU to achieve a final solution of 1 mg/mL BrdU. Store at 4 for one month.
3. **4% PFA**
**Caution:** Preparation of PFA is hazardous. Avoid skin contact and inhalation. Use appropriate PPE and a chemical fume hood.
  Mix 4 g of PFA and 100 mL of 1× PBS in a media storage bottle containing a stir bar. Add drops of 2 M NaOH while the solution is stirring until a pH of approximately 7.4 has been reached or the PFA has completely dissolved. Please note the solution may become more acidic as PFA dissolves and the addition of more NaOH may be necessary. Place the bottle on a hot plate heated to 85 for approximately 20 min or until there are no visible particles. Allow the solution to cool at 4 . Add 1 M HCl drops until the solution reaches a pH of 6.9. Store at 4 for one week.
4. **FACS buffer with 4% FBS**
  Add 4 mL of FBS to 96 mL of 1× PBS. Store at 4 for one month.
5. **0.4% Triton X-100**
  Add 4 mL of Triton X-100 to 96 mL of 1× PBS. Store at 4 for several months.
6. **0.1 M HCl**
  Add 1.5 mL of 1 M HCl to 13.5 mL of MilliQ water for a final concentration of 0.1 M HCl. Store at room temperature for several months.

**Laboratory supplies**

1. 10 mm Petri dish (VWR, catalog number: 10799-192)
2. 90 mm Petri dish (Sigma-Aldrich, catalog number: P10903)
3. General lab supplies (pipettes, tips, tubes, etc.)
4. 5 mL Falcon round-bottom Polystyrene test tubes with 70 μm cell strainer (Fisher, catalog number: 08-771-23)

## Equipment

1. Dumont #7 curved forceps (Fine Science Tools, catalog number: 11274-20)
2. Dumont #5 forceps (Fine Science Tools, catalog number: 11251-10)
3. Spring scissors (Fine Science Tools, catalog number: 15000-00)
4. Scalpel blades #11 (Fine Science Tools, catalog number: 10011-00)
5. Scalpel handle (Fine Science Tools, catalog number: 10003-12)
6. Corning^®^ LSE^TM^ digital water bath, 6 L, 120 V (Corning, catalog number: 6783)
7. Fisherbrand^TM ^analog hotplate stirrer (Fisher, catalog number: FB30786160)
8. pH indicator strips (Sigma-Aldrich, catalog number: 1095350001)
9. VWR^®^ water jacketed CO incubator (VWR, catalog number: 10810-744)
10. Pipet-Aid (Corning, catalog number: 07-202-350)
11. Centrifuge (Thermo Fisher Scientific, model: Heraeus Multifuge X3)
12. Eppendorf benchtop centrifuge (for microcentrifuge tubes at room temperature)
13. Benchtop vortex
14. Bright-Line counting chamber, which includes slides (VWR, catalog number: 100503-092)
*Note: Alternatively, an automated cell counter can be used for this protocol.*
15. BD LSRFortessa^TM^ cell analyzer (BD Biosciences)

## Software and datasets

1. FlowJo^TM^ software v10.10 (BD Life Sciences) was used for flow cytometry data analysis

## Procedure

- **Eyedrop application**
  1. Treat adult mice (3–6 month old) with eyedrops containing either 1% DMSO and 1 mg/mL BrdU or 1 mM PNU-282987 and 1 mg/mL BrdU for 28 days. Detailed instructions on how to apply eyedrops can be found in Linn et al. [11]. Briefly, the mouse is immobilized by scuffing the skin over their neck. One drop of DMSO/BrdU or DMSO/PNU-282987/BrdU eyedrop solution is placed on the bulbar conjunctiva of the right eye using a transfer pipette positioned upward by twisting the restrainer’s hand. The drop should cover the entire eye and should sit on the top of the eye for 1–2 s. Next, the restrainer twists their hand so that the left eye is positioned upward. A drop of DMSO/BrdU or DMSO/PNU-282987/BrdU eyedrop solution is placed on the bulbar conjunctiva of the left eye and allowed to sit for 1–2 s.
- **Retina harvesting**
  1. After euthanasia by carbon dioxide asphyxiation, place Dumont #7 curved forceps around the posterior aspect of the eyeball near the optic nerve. Apply gentle pressure and lift the eyeball out of the cavity. Place in a 10 mm Petri dish filled with 3 mL of ice-cold 1× PBS (Figure 1A).
  2. Grasping the sclera with Dumont #5 forceps, move the eye out of the Petri dish. Create a puncture hole using a scalpel along the corneal equator (Figure 1B and 1C).
  3. Hold the edge of the puncture hole with Dumont #5 forceps and place the eye back into the Petri dish. Insert Spring scissors into the puncture hole to create a circumferential incision along the corneal equator (Figure 1D).
  4. Using a second pair of Dumont #5 forceps, begin to peel away the cornea and the iris from the posterior eye cup. Grasping the optic nerve with Dumont #5 forceps to generate traction may be necessary at this step (Figure 1E and 1F).
  5. Once the cornea and iris are peeled away, the lens will be fully revealed. Remove the lens from the posterior eye cup with Dumont #5 forceps (Figure 1G).
  6. Evert the posterior eyecup with Dumont #5 forceps to prolapse the retina (Figure 1H).
  7. Holding the sclera with Dumont #5 forceps, remove the retina from the posterior eye cup with Spring scissors (Figure 1I). Remove any remaining, black-pigmented retinal epithelium with Spring scissors.
  8. Rinse the isolated retina by submerging it in a new 10 mm Petri dish filled with 3 mL of ice-cold 1× PBS (Figure 1J).
    **Figure 1.**
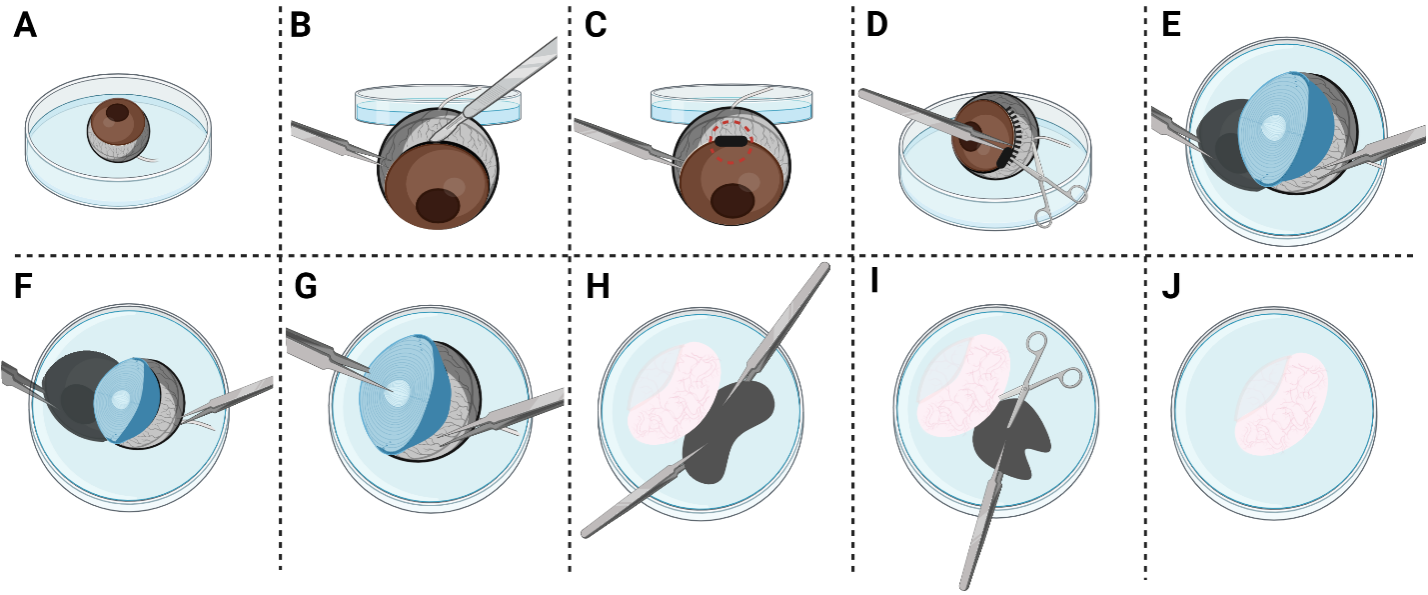
Illustration demonstrating retina dissection. (A) Submerge the enucleated mouse eye in ice-cold 1× PBS in a 10 mm Petri dish. (B) Using Dumont #5 forceps, grasp the sclera and move the eye outside of Petri dish. While securing the eye in place with Dumont #5 forceps, create a scleral puncture using a scalpel along the corneal equator. (C) The black rectangle surrounded by the dotted circle depicts where the puncture wound should be placed. (D) Using Dumont #5 forceps, grasp the edge of the puncture wound to place the eye back into the dish. Insert Spring scissors into the puncture wound and create a circumferential incision along the corneal equator. (E) Using a second pair of Dumont #5 forceps, start peeling away the cornea and the iris from the posterior eye cup. (F) Grasp the optic nerve to generate traction. (G) Remove the lens while grasping the posterior eyecup with Dumont #5 forceps. (H) Prolapse the retina by everting the posterior eyecup with Dumont #5 forceps. (I) Secure the posterior eyecup in place with Dumont #5 forceps. Detach the retina from the posterior eyecup with Spring scissors. If any black pigmented retinal pigment epithelium remains, gently remove it with Spring scissors. (J) View depicting the isolated retina after dissection. After dissection, the isolated retina should be rinsed by submerging it in a new Petri dish filled with ice-cold 1× PBS.
- **Retina dissociation**
  1. Follow the manufacturer’s instructions for preparing papain, DNase I, and ovomucoid inhibitor to yield the proper concentrations.
  2. Transfer the four retinae using Dumont #5 forceps into a 1.5 mL microcentrifuge tube filled with 500 μL of papain that has been warmed to 37 for at least 10 min in a water bath.
  3. Mince the retinae into eight pieces using Spring scissors.
  4. Add 25 μL of 1 mg/mL DNase I to papain.
  5. Close the microcentrifuge tube lid and place it in an incubator heated to 37 on a rocker for constant agitation for 60 min.
*Note: Optimal papain incubation time was determined in a separate experiment to investigate which incubation time yielded the highest number of living cells (Figure S1).*
  6. Pour the solution into a 90 mm Petri dish. Use warm EBSS to rinse out any remaining retinal tissue.
  7. Triturate the tissue with a 10 mL pipette at least five times or until the solution appears cloudy and there are no large pieces of retinal tissue remaining. Transfer contents to a 15 mL conical tube.
**Caution:** The introduction of bubbles during trituration should be avoided. Bubbles have a high surface tension that can cause lysis.
  8. Centrifuge at 300× *g* for 5 min.
  9. While contents are centrifuging, create a resuspension solution in a 15 mL conical tube containing 2.7 mL of EBSS, 300 μL of ovomucoid inhibitor solution, and 150 μL of DNase I.
  10. Remove the supernatant. Using a 10 mL pipette, transfer the entire resuspension solution created in step C9 to the conical tube containing the cell pellet and resuspend the cell pellet by triturating it 1–2 times.
  11. Prepare discontinuous density gradient. In a new 15 mL conical tube, transfer 5 mL of ovomucoid inhibitor solution prepared in step C1 using a 10 mL pipette. Next, using a 10 mL pipette, add the entirety of the cell suspension created in step C10 on top of the 5 mL of albumin-ovomucoid solution to create a two-phase solution.
**Caution:** It is important to avoid mixing the two-phase solution. We have found that the best way to avoid mixing is to tilt the Pipet-Aid containing the cell suspension horizontally and release the liquid on the “slow” setting.
  12. Centrifuge at 70× *g* for 6 min.
*Note: A flow cytometry plot depicting a freshly dissociated retina is represented in Figure 2.*
    **Figure 2.**
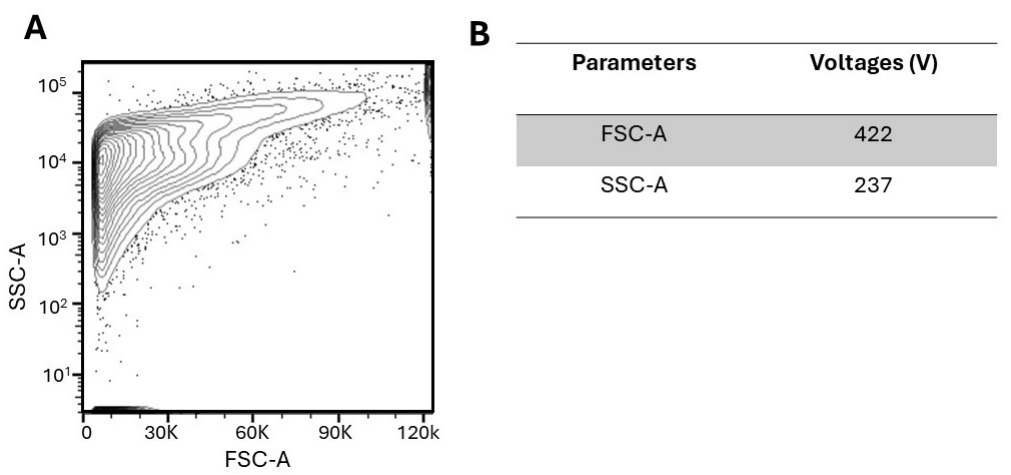
Unfixed and unpermeabilized dissociated retinae. (A) Plot depicting unfixed and unpermeabilized dissociated retinae with no fluorescent antibodies added. (B) Parameters include FSC (forward scatter; size) and SSC (side scatter; granularity). The corresponding voltages were implemented to detect an optimal signal above the noise and resolve positive and negative populations.
- **Fixation and permeabilization**
  1. Discard supernatant and resuspend the cell pellet by triturating it 3–5 times in 2 mL of 4% PFA using a 10 mL pipette. Let this sit for 15 min at room temperature for fixation.
  2. Centrifuge at 500× *g* for 5 min.
  3. Discard supernatant in hazardous waste and wash cell pellet by resuspension, triturating it 1–2 times in 2 mL of FACS buffer.
  4. Centrifuge at 500× *g* for 5 min.
  5. Discard the supernatant and resuspend the cell pellet by triturating it 3–5 times in 2 mL of 0.1 M HCl that has been prewarmed to 37 using a 10 mL pipette. Incubate at 37 in a warmer for 15 min.
  6. Centrifuge at 500× *g* for 5 min.
  7. Discard the supernatant and resuspend the cell pellet by triturating it 3–5 times in 2 mL of 0.4% Triton-X 100 using a 10 mL pipette. Let this sit for 15 min at room temperature for permeabilization.
*Note: A flow cytometry plot depicting a fixed and permeabilized retina is represented in Figure 3.*
    **Figure 3.**
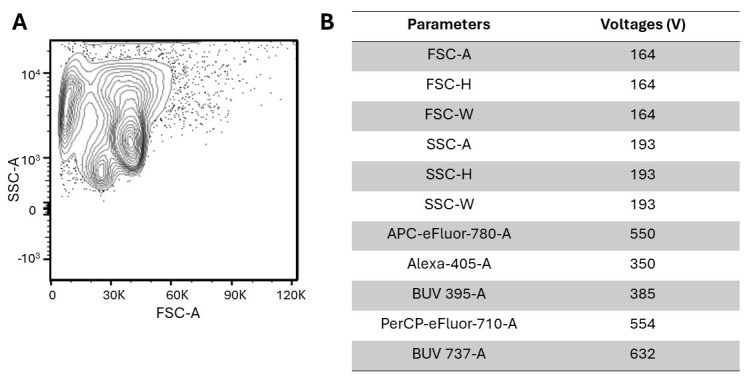
Fixed and permeabilized dissociated retinae. (A) Plot depicting fixed and permeabilized dissociated retinae with no fluorescent antibodies added. The light-scatter properties of cells that have been fixed and permeabilized are typically altered, as evidenced by comparing Figure 2 and Figure 3. However, after excluding debris with gating, the two plots are comparable. (B) Parameters include FSC (forward scatter; identifies size), SSC (side scatter; identifies granularity), APC-eFluor-780 (Thy1.2), Alexa-405 (rhodopsin), BUV 395 (vimentin), PerCP-eFluor-710 (BrdU), and BUV 737 (Ki67). The corresponding voltages were implemented to detect an optimal signal above the noise and resolve positive and negative populations.
  8. While incubating, collect 20 μL of the suspension and count cells using an automated cell counter or manually using a hemocytometer.
*Note: Cell counts are approximately 1 × 10^6^ per two retinas.*
  9. Centrifuge at 500× *g* for 5 min.
  10. Remove the supernatant. Add 20 μL of Fc block and 50 μL of BD Horizon Brillant Stain Buffer to all experimental tubes.
  11. Incubate for 15 min at room temperature.
  12. Add 2 mL of FACS buffer using a 10 mL pipette and centrifuge at 500× *g* for 5 min.
    Discard the supernatant and resuspend cells in 1 mL of FACS buffer by triturating it 1–2 times using a 1 mL glass pipette. Remove suspension from 15 mL conical using a 1 mL glass pipette and add it to a 1.5 mL microcentrifuge tube. The following groups should be created: A) single-stain compensation beads for each color in separate 1.5 mL microcentrifuge tubes (Table 2); B) untreated/unstained cells (Table 3); C) DMSO control (Table 3); and D) PNU-282987 treated (Table 3). Our panel included the following controls: A) compensation beads + BUV-395 only, compensation beads + APC-Efluor-780 only, compensation beads + Alexa-405 only, compensation beads + PerCP-Efluor-710 only, compensation beads + BUV-737 only; B) untreated/unstained cells; and the following experimental groups: C) DMSO control cells with Vimentin_Strep-BUV-395; Thy1.2_APC-eFluor-780; Rho_Alexa-405; BrdU_PerCP-eFluor-710; Ki67_BUV-737; and D) PNU-282987-treated cells with Vimentin_Strep-BUV-395; Thy1.2_APC-eFluor-780; Rho_Alexa-405; BrdU_PerCP-eFluor-710; Ki67_BUV-737.
*Notes:*
    - *Compensation controls are necessary to set instrument voltages and compensate samples. Compensation controls must be repeated for every experiment. In the case of the retina, we found beads are the most appropriate material for compensation as compared to retina cells.*
    - *Please note that FMOs are utilized to determine positive vs. negative gates for the fluorophores included in the panel. FMOs need to be run during panel setup. An example of the FMO panels used in this experiment can be found in Figure S2.*
      **Table 2.** Compensation controls used in this protocol

Amount of compensation beads (per tube)	Cell type	Stain	Amount of antibody added
1 drop	Unstained	-	-
1 drop	Müller glia	Vimentin_Strep-BUV-395	1 μL
1 drop	Retinal ganglion cells	Thy1.2_APC-eFluor-780	1 μL
1 drop	Rod photoreceptors	Rho_Alexa-405	1 μL
1 drop	Proliferating cells	Ki67_BUV-737	1 μL
1 drop	Mitotically active cells	BrdU_PerCP-eFluor-710	1 μL
      **Table 3.** Experimental groups used in this protocol

Experimental groups	# retinae (per tube)	Approximate cell count	Cell type	Stain
Untreated/unstained	4	~2 M	-	-
DMSO control	4	~2 M	Müller glia, retinal ganglion cells, rod photoreceptors, proliferating cells, mitotically active cells	Vimentin_Strep-BUV-395, Thy1.2_APC-eFluor-780, Rho_Alexa-405, Ki67_BUV-737, BrdU_PerCP-eFluor-710
PNU-282987 treated	4	~2 M	Müller glia, retinal ganglion cells, rod photoreceptors, proliferating cells, mitotically active cells	Vimentin_Strep-BUV-395, Thy1.2_APC-eFluor-780, Rho_Alexa-405, Ki67_BUV-737, BrdU_PerCP-eFluor-710
- **Staining for flow cytometry**
  1. Compensation beads
    - Vortex compensation beads.
    - Add 1 drop of compensation beads into a 1.5 mL microcentrifuge tube.
    - Add 1 μL of antibody/stain to each tube.
    - Incubate on ice for 30 min.
    - Add 1 mL of FACS buffer to each tube.
    - Centrifuge at 150× *g* for 5 min.
    - Remove supernatant and resuspend the pellet by triturating it 1–2 times in 500 μL of FACS buffer using a p1000 pipette.
    - Transfer the sample into a 5 mL Falcon round-bottom polystyrene test tube by passing it through the 70 μm cell strainer.
  2. Vimentin
    - Add the appropriate amount of antibody (see Table 1) that is used to label Müller glia using vimentin to the 1.5 mL microcentrifuge tube containing the cell pellet resuspended in 1 mL of FACS buffer. Briefly vortex the sample.
    - Allow this to sit for 30 min at room temperature.
    - Using a p1000 pipette, add 500 μL of FACS buffer.
    - Centrifuge at 150× *g* for 5 min.
    - Remove supernatant and resuspend cells by triturating it 1–2 times in 1 mL of FACS buffer using a p1000 pipette.
    - After briefly vortexing the streptavidin BUV-395, add it to the cell suspension at a concentration of 2 µL per 1 × 10^6^ cells.
    - Allow this to sit for 30 min at room temperature.
    - Centrifuge at 150× *g* for 5 min.
    - Remove supernatant and triturate the cell pellet 1–2 times in 500 μL of FACS buffer using a p1000 pipette
    - Transfer the sample into a 5 mL Falcon round-bottom polystyrene test tube by passing it through the 70 μm cell strainer.
  3. Rhodopsin
    - Prepare the rhodopsin antibody solution by combining 5 μL of mouse α Rhodopsin IgG, 5 μL of Zenon^TM^ mouse IgG labeling reagent (Component A), and ≤ 20 μL of 1× PBS in a separate 1.5 mL microcentrifuge tube.
    - Incubate in the dark for 5 min at room temperature.
    - Add 5 μL of Zenon^TM^ blocking reagent (Component B) to the antibody solution.
    - Incubate in the dark for 5 min at room temperature.
    - Add the appropriate amount of antibody (see Table 1) that is used to label Müller glia using vimentin to the 1.5 mL microcentrifuge tube containing the cell pellet resuspended in 1 mL of FACS buffer. Briefly vortex the sample.
    - Incubate in the dark at room temperature for 30 min.
    - Add 500 μL of FACS buffer.
    - Centrifuge at 150× *g* for 5 min.
    - Remove the supernatant and resuspend the cell pellet by triturating it 1–2 times in 500 μL of FACS buffer using a p1000 pipette.
    - Transfer the sample into a 5 mL Falcon round-bottom polystyrene test tube by passing it through the 70 μm cell strainer.
  4. All other antibodies used
    - Add the appropriate volume of each antibody (see Table 1) to the 1.5 mL microcentrifuge tube containing the cell pellet resuspended in 1 mL of FACS buffer. Briefly vortex the sample.
*Note: Optimal antibody concentrations were determined using a stain index.*
    - Incubate in the dark for 30 min.
    - Add 500 μL of FACS buffer.
    - Centrifuge at 150× *g* for 5 min.
    - Remove supernatant and resuspend the cell pellet by triturating it 1–2 times in 500 μL of FACS buffer using a p1000 pipette.
    - Transfer the sample into a 5 mL Falcon round-bottom polystyrene test tube by passing it through the 70 μm cell strainer.

## Data analysis

**Figure 4.**
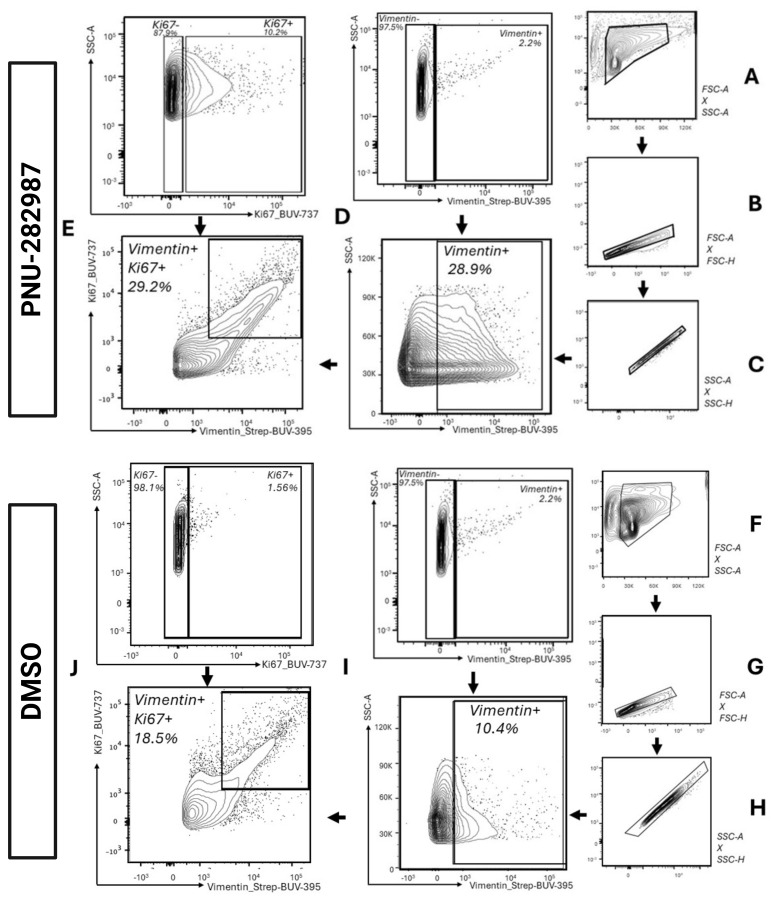
Representative gating strategy used for gating vimentin+ and Ki67+ cells after 28 days of DMSO and PNU-282987 treatment. (A) and (F) A gate is made around the main cell population to exclude debris (SSC-A vs. FSC-A). (B–C) and (G–H) Singlet gates are drawn using FSC-H vs. FSC-A and SSC-H vs. SSC-A. (D) and (I) Vimentin+ cells are gated using FMOs to determine the cell population positive for vimentin (FSC-A vs. BUV-395-A). Back-gating figure for vimentin, displaying vimentin+ cells in the parent populations, is depicted in Figure S3. (E) and (J) Vimentin+ cells that are also labeled for the proliferation marker Ki67 are gated around. FMOs were utilized to determine positive cell populations (BUV-737-A vs. BUV-395-A).

1. Selecting cells and excluding debris:
  Use the Forward Scatter Area vs. Side Scatter Area (FSC-A vs. SSC-A) density plot to gate for the cell population while excluding cellular debris. Cellular debris is eliminated from the analysis by using a gate that excludes signals with a low level of forward scatter, usually aggregated at the bottom left corner (Figure 4A and Figure 4F).
2. Selecting singlets:
  Use the Forward Scatter Area vs. Forward Scatter Height (FSC-A vs. FSC-H) density plot to gate for singlets (single cells) usually aggregated on the 45° diagonal line (Figure 4B and Figure 4G). Next, use the Side Scatter Area vs. Side Scatter Height (SSC-A vs. SSC-H) density plot to further refine the single-cell population aggregated on the 45° diagonal line (Figure 4C and Figure 4H).
3. Identifying populations with specific phenotype markers:
  For each color of interest, select the positive population as identified in FMO (Figure 4D and Figure 4I). Then, gate on the proliferating population as identified in FMO (Figure 4E and Figure 4J).

## Validation of protocol

**Figure 5.**
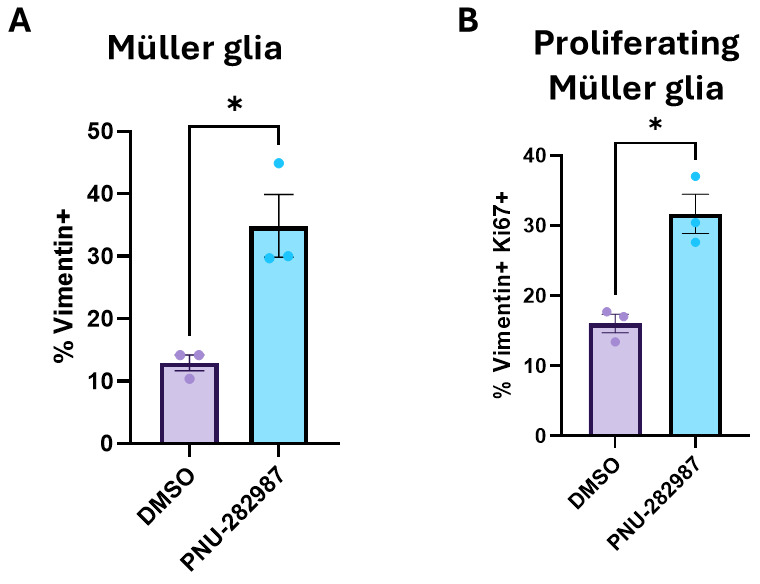
Müller glia and proliferating Müller glia are increased in PNU-282987-treated samples. Adult (3–6 month old) mice were treated with 1× PBS eyedrops containing 1% DMSO and 1 mg/mL BrdU or 1 mM PNU-282987 and 1 mg/mL BrdU for 28 days. Subsequently, retinae were dissociated and processed for analysis by flow cytometry. (A) Graph showing the percent increase of vimentin+ Müller glia cells in DMSO vs. PNU-282987-treated samples. (B) Graph showing the percent increase of vimentin+Ki67+ Müller glia in DMSO vs. PNU-282987-treated samples. Total retinae: DMSO n = 12; PNU-282987 n = 12. Results are based on three independent experiments with retinae from mice pooled in each of the three experiments. Statistics: unpaired *t*-test with Welch’s correction; *p < 0.05.

Representative results from 28 days of DMSO and PNU-282987 treatment are presented in Figure 4. Quantitative results are shown in Figure 5.

## General notes and troubleshooting

The discontinuous density gradient where the two layers of the gradient are visible, created in the dissociation protocol, is essential for a high cell yield. We have found that the best way to create a proper gradient is to turn the Pipet-Aid speed setting to slow, hold the Pipet-Aid horizontally so that the opening of the serological pipette is touching the inside of the tube, and slowly release the suspension so that it delicately layers.

Gentle or overly vigorous trituration of retinal tissue after incubation with papain during the dissociation process will result in a low yield of cells. Additionally, using a pipette that has a small opening could shear the cells. Therefore, we have found the tissue must be triturated at least five times with a 10 mL serological pipette so that the resulting solution is cloudy. However, if the resulting cell numbers are low with five triturations, lowering the number of triturations could result in a higher yield.

## Supplementary information

The following supporting information can be downloaded here:

1. Figure S1. Sixty minutes is the optimal time for retinae papain digestion.
2. Figure S2. Fluorescence minus one(FMO) for photoreceptors, retinal ganglion cells, and mitotically active cells.
3. Figure S3. Back-gating for vimentin+ Müller glia.
